# Clusterin facilitates apoptotic cell clearance and prevents apoptotic cell-induced autoimmune responses

**DOI:** 10.1038/cddis.2016.113

**Published:** 2016-05-05

**Authors:** P Cunin, C Beauvillain, C Miot, J-F Augusto, L Preisser, S Blanchard, P Pignon, M Scotet, E Garo, I Fremaux, A Chevailler, J-F Subra, P Blanco, M R Wilson, P Jeannin, Y Delneste

**Affiliations:** 1 Université d'Angers, Angers, France; 2 Unit 892, Inserm, CHU d'Angers, Angers, France; 3 CNRS, Unit 6299, Angers, France; 4 LabEx IGO "Immuno-Graft-Onco", Angers, France; 5 Laboratoire d'Immunologie et d'Allergologie, CHU Angers, Angers, France; 6 Service de Néphrologie-Dialyse-Transplantation, CHU Angers, Angers, France; 7 Université Victor Segalen, Bordeaux, France; 8 CNRS Unit 5164, Bordeaux, France; 9 CHU Bordeaux, Bordeaux, France; 10 School of Biological Sciences, Illawarra Health and Medical Research Institute, University of Wollongong, Wollongong, New South Wales, Australia

## Abstract

Clusterin (Clu), an extracellular chaperone, exhibits characteristics of soluble innate immunity receptors, as assessed by its ability to bind some bacteria strains. In this study, we report that Clu also binds specifically to late apoptotic cells but not to live, early apoptotic, or necrotic cells. Histones, which accumulate on blebs during the apoptotic process, represent privileged Clu-binding motifs at the surface of late apoptotic cells. As a consequence, Clu potentiates, both *in vitro* and *in vivo*, the phagocytosis of late apoptotic cells by macrophages. Moreover, the increased phagocytosis of late apoptotic cells induced by Clu favors the presentation and cross-presentation of apoptotic cell-associated antigens. Finally, we observed that, in a model of apoptotic cell-induced autoimmunity, and relative to control mice, Clu^−/−^ mice develop symptoms of autoimmunity, including the generation of anti-dsDNA antibodies, deposition of immunoglobulins and complement components within kidneys, and splenomegaly. These results identify Clu as a new molecule partner involved in apoptotic cell efferocytosis and suggest a protective role for Clu in inflammation and autoimmune diseases.

Clusterin (Clu), also known as apolipoprotein J, is a soluble 80-kDa disulfide-linked heterodimeric glycoprotein which is highly conserved during evolution and among mammals.^1^ Clu is abundant in physiological fluids (concentrations ranging from 100 to 300 μg/ml in human serum)^2, 3, 4^ and is induced in response to a wide variety of tissue injuries. Clu has chaperone activity and is a functional homolog to small heat-shock proteins.^5, 6^ It binds hydrophobic domains of numerous non-native proteins and targets them for receptor-mediated internalization and lysosomal degradation. Clu also interacts with a broad spectrum of molecules (such as lipids, components of the complement system, amyloid-forming proteins, immunoglobulins)^7, 8^ and has been suggested to regulate several functions, such as complement activity, cell–cell and cell–substratum interactions, and cell proliferation/survival.^1^ In various diseases, an accumulation of Clu has been reported in the injured organs.^9, 10^

Clu also interacts with different immune molecules; however, its potential role in immune responses remains unclear. Clu binds to some bacteria (*Staphylococcus aureus* and some *Staphylococcus epidermidis* strains) and bacterial proteins (such as the *Streptococcus pyogenes* extracellular protein SIC),^11, 12, 13^ suggesting that it may modulate antimicrobial responses. Moreover, Clu limits the severity of induced autoimmune myocarditis^14^ and pancreatitis.^15^ Finally, the levels of circulating Clu in systemic lupus erythematosus,^16^ as well as the expression of Clu mRNA in the synovium of rheumatoid arthritis patients, are decreased.^17^

Phagocytosis of dying cells, a process called efferocytosis, is a complex mechanism that involves (i) exposure at the apoptotic cell surface of phosphatidylserine (PS) and membrane molecules that are altered during the apoptotic process,^18^ and (ii) endocytic receptors expressed by phagocytes, such as members of the scavenger receptor family,^19^ vitronectin receptors,^20^ Fc receptors,^21^ MER,^22^ TIM-1 and TIM-4,^23^ and CD91.^24^ Soluble receptors (also called opsonins), such as C1q, mannose-binding lectin (MBL),^25^ and milk fat globule-EGF factor 8 (MFG-E8),^26^ bind to apoptotic cells and act as bridging molecules to favor their internalization by phagocytes. In a non-inflammatory environment, a rapid and efficient clearance of apoptotic cells maintains immune homeostasis and avoids the initiation of autoimmune responses.^27, 28^ In contrast, a dysfunction in the clearance of apoptotic cells may result in the release of danger molecules (referred to as danger-associated molecular patterns) that may favor the initiation of autoimmune responses.^27, 28^

The pivotal role played by soluble molecules in apoptotic cell clearance and, consequently the outcome of immune responses to apoptotic cell antigens, has been clearly evidenced in opsonin-deficient mice. For example, mice deficient in MFG-E8, C1q, or serum amyloid P component (SAP) show impaired clearance of apoptotic cells and develop a lupus-like disease characterized by elevated levels of autoantibodies and glomerulonephritis.^29, 30, 31^

Some soluble innate immunity receptors involved in microbial recognition have also been implicated in apoptotic cell clearance.^19^ As Clu binds to microbial moieties,^9, 10, 11^ we investigated whether Clu might also mediate apoptotic cell clearance by phagocytes.

## Results

### Clu binds to late apoptotic cells

**Figure 1:**
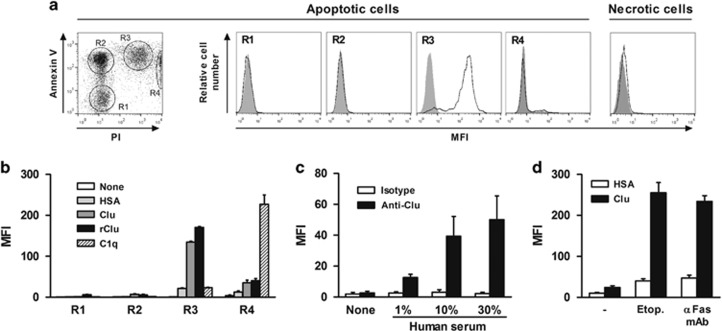
Clu binds to late apoptotic cells. (**a**) Left panel, analysis of spontaneous apoptosis of human neutrophils after culture in 1% FCS culture medium and staining with PI and APC-labeled Ann V. Flow cytometry analysis allowed to identify four populations corresponding to viable (Ann V^−^ PI^−^; R1), early apoptotic (Ann V^+^ PI^−^; R2), late apoptotic (Ann V^+^ PI^+^; R3), and necrotic cells (Ann V^+/−^ PI^high^; R4), respectively. Middle panels, dying neutrophils were incubated or not with 1 μM OG-Clu; the binding of Clu to R1–R4 populations was evaluated by flow cytometry. Right panel, binding of OG-Clu to heat-induced necrotic neutrophils. Results are representative of five independent experiments. (**b**) Neutrophils, at different apoptosis stages (R1–R4), were incubated with 1 μM OG-HSA, OG-Clu, OG-rClu, or OG-C1q. Binding was analyzed by flow cytometry. Results are expressed in MFI values, mean±S.E.M., *n*=5. (**c**) Late apoptotic neutrophils were incubated with increasing concentration of human serum and stained with an anti-Clu or isotype-matched mAb. Bound Abs were detected by flow cytometry using a FITC-labeled anti-mouse Ig Ab. Results are expressed in MFI values, mean±S.E.M., *n*=4. (**d**) Binding of OG-Clu to late apoptotic Jurkat T cells. The apoptosis was induced with 20 μg/ml etoposide or 20 ng/ml anti-FAS mAb. Results are expressed in MFI values, mean±S.E.M., *n*=4

We first evaluated the ability of recombinant human Clu to bind to spontaneously dying human neutrophils. As previously described,^32^ four populations can be distinguished by flow cytometry, based on annexin V (Ann V) and propidium iodide (PI) staining (Figure 1a, left panel): viable (Ann V^−^ PI^−^, corresponding to R1), early apoptotic (Ann V^+^ PI^−^; R2), late apoptotic (Ann V^+^ PI^+^; R3), and secondary necrotic cells (Ann V^+/−^ PI^high^; R4). Results showed that Oregon Green 488 (OG)-labeled Clu (OG-Clu) binds to late apoptotic (R3) and, to a low extent, to secondary necrotic neutrophils (R4), but not to viable (R1) and early apoptotic (R2) neutrophils (Figure 1a, middle panels). A low binding of OG-Clu was also detected to heat-induced necrotic neutrophils (Figure 1a, right panel). Similar binding profiles were obtained using purified and recombinant Clu revealed by a FITC-labeled anti-Clu mAb (Figure 1b). As control,^25, 33^ OG-C1q binds preferentially to late apoptotic and secondary necrotic cells (Figure 1b). No binding of the control protein OG-HSA (human serum albumin) was observed on dying cells (Figure 1b).

We next investigated whether Clu present in human serum also binds to late apoptotic cells. Dying neutrophils were incubated with human serum and bound Clu was detected using a FITC-labeled anti-Clu mAb. Results showed that Clu present in human serum binds to late apoptotic cells and that the level of binding was dependent on the concentration of serum used (Figure 1c); no binding of the anti-Clu mAb on late apoptotic cells was observed in the absence of serum (Figure 1c), demonstrating that intracellular Clu does not translocate to the surface of dying cells during the apoptotic process. Finally, we observed that Clu also binds to (i) late apoptotic Jurkat cells (induced by etoposide or an anti-Fas mAb) (Figure 1d), (ii) apoptotic murine thymocytes (Supplementary Figure S1A), and (iii) tumor cells either irradiated or treated with etoposide (Supplementary Figure S1B), demonstrating that the binding of Clu to late apoptotic cells is not dependent on the cell type or on the apoptosis-inducing signal.

### Clu binds to histones expressed at the surface of late apoptotic cells

**Figure 2:**
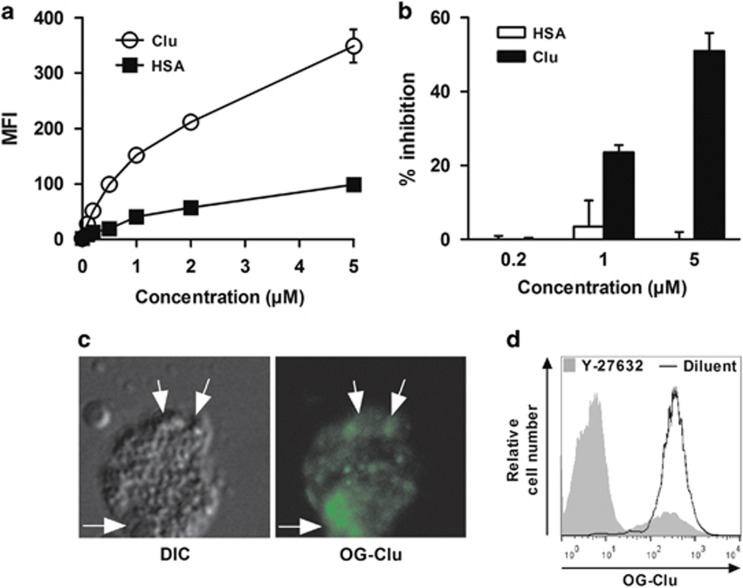
Clu-binding elements are localized on blebs. (**a**) Late apoptotic neutrophils were incubated with increasing concentrations of OG-Clu or OG-HSA; the binding was analyzed by flow cytometry. Results are expressed in MFI values, mean±S.E.M., *n*=5. (**b**) Late apoptotic neutrophils were incubated with the indicated concentrations of unlabeled Clu or HSA before addition of 1 μM OG-Clu; the binding of OG-Clu was analyzed by flow cytometry. Results are expressed as the percentages of inhibition, mean±S.E.M., *n*=5. (**c**) Differential interference contrast (DIC; left panel) and confocal fluorescence (right panel) microscopic images of late apoptotic neutrophils incubated with OG-Clu. Images are representative of two independent experiments. White arrows, blebs; original magnification, × 630. (**d**) Neutrophils were incubated or not with 50 μg/ml Y-27632, prior to apoptosis induction. The binding of OG-Clu was evaluated by flow cytometry. Histograms are representative of three independent experiments

The binding of OG-Clu to late apoptotic neutrophils is dose dependent, partly saturable (Figure 2a), and is inhibited in a dose-dependent manner, by unlabeled Clu, but not by HSA (Figure 2b). Fluorescence microscopy revealed an intense staining on bleb-like structures (Figure 2c). This binding was reduced on apoptotic neutrophils (Figure 2d), which were left to die in the presence of Y-27632, an inhibitor of membrane blebbing.^34^ These observations suggested the presence of Clu-binding elements at the surface of late apoptotic cells.

**Figure 3:**
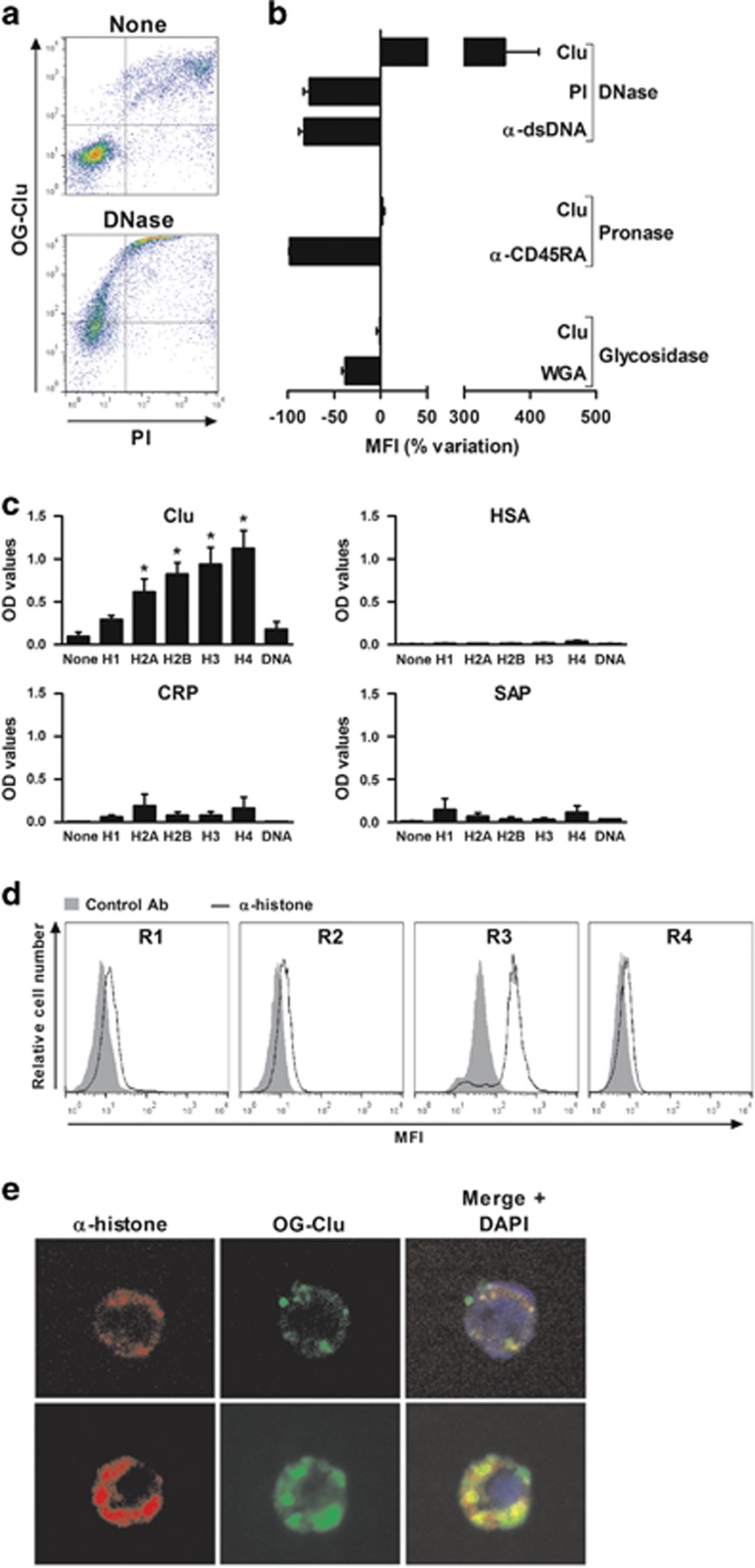
Clu binds to histones expressed on late apoptotic cells. (**a**) Late apoptotic Jurkat T cells were treated with 500 μg/ml DNase prior to incubation with 1 μM OG-Clu. DNase efficiency was assessed by PI staining. Dot plots are representative of four independent experiments. (**b**) Binding of OG-Clu to apoptotic Jurkat T cells treated with 500 μg/ml DNase, 100 μg/ml pronase, or 100 μg/ml glycosidase. As controls of DNase, pronase, and glycosidase efficiency, binding of PI, anti-dsDNA mAb, anti-CD45RA mAb, and FITC-labeled WGA was analyzed. Results are expressed as a percentage of variation of the binding of Clu, PI, anti-dsDNA mAb, anti-CD45RA mAb, and WGA on treated compared with non-treated cells, mean±S.E.M., *n*=4. (**c**) H1, H2A, H2B, H3, and H4 histone subunits and genomic human DNA were immobilized on a 96-well plate. Binding of biotinylated-HSA, -Clu, -CRP, and -SAP to histone subunits and DNA was determined by enzyme-linked immunosorbent assay. Results are expressed in OD values, mean±S.E.M.; *n*=5. *P<0.05 compared to none (Mann-Whitney test). (**d**) Apoptotic neutrophils were labeled with anti-H2/H3/H4 or isotype control mAbs, before incubation with a FITC-labeled anti-mouse IgG Ab. Live (R1), early apoptotic (R2), late apoptotic (R3), and necrotic neutrophils (R4) were discriminated by flow cytometry using Ann V and PI staining. Results are representative of one out four experiments. (**e**) Apoptotic neutrophils were incubated with OG-Clu in the presence of anti-H4 (upper panels) or anti-H2/H3/H4 mAbs (lower panels) prior to incubation with a PE-labeled anti-mouse IgG Ab. Presence of OG-Clu and histones on cell surface was assessed by confocal microscopy. DNA was stained with DAPI. Original magnification, × 630. Images are representative of one of the four experiments

The apoptotic process is accompanied by cell surface alterations, such as PS externalization, membrane relocalization of intracellular components, and oxidation of membrane molecules;^35, 36^ these motifs act as 'eat-me' molecules. In an attempt to characterize the nature of the Clu-binding motif(s), we evaluated whether a treatment with DNase, glycosidases, or pronase may modulate the binding of Clu to late apoptotic cells. DNase strongly upregulated the binding of Clu to apoptotic Jurkat cells (increase of 362±51% mean±S.E.M., *n*=4; Figures 3a and b); as a control for DNase efficiency, the staining with PI and the binding of an anti-dsDNA mAb was lower on DNase-treated than on non-treated apoptotic cells (Figures 3a and b). In contrast, pronase or glycosidases did not modulate the binding of Clu to apoptotic cells (Figure 3b); as enzyme controls, pronase and glycosidases decreased the binding on late apoptotic cells of anti-CD45RA mAb^37^ and of the lectin wheat germ agglutinin (WGA),^38^ respectively (Figure 3b). The treatment of late apoptotic cells with DNAse and pronase strongly accelerated the generation of remnant cells (data not shown), excluding evaluating the binding of Clu on apoptotic cells treated with the two enzymes. Finally, a negligible amount of Clu bound to erythrocytes treated with ionomycin (Supplementary Figure S2A), which induces phospholipid externalization,^39^ making unlikely a role for externalized lipids in the binding of Clu. Accordingly, no binding of Clu was detected on immobilized lipids (Supplementary Figure S2B).

The fact that DNAse increased the binding of Clu to late apoptotic cells suggested that DNA may sterically mask Clu-binding elements. As genomic DNA is associated with histones, we suspected that histones might represent Clu-binding elements. A solid-phase binding assay showed that Clu binds to immobilized H2A, H2B, H3, and H4, and, to a lower extent, to the linker subunit H1 (Figure 3c). As control,^40, 41^ C-reactive protein (CRP) and SAP, but not HSA, also bind to histones (Figure 3c). No binding of Clu to immobilized dsDNA was observed (Figure 3c); as a control, immobilized dsDNA was recognized by anti-dsDNA Abs (data not shown).

We then confirmed that histones, translocated to the surface of apoptotic cells, represent Clu-binding elements. As reported,^36^ histones can be detected at the surface of late apoptotic neutrophils but not on viable, early apoptotic, and necrotic neutrophils (Figure 3d). Interestingly, DNAse increased the binding of an anti-histone Ab (Supplementary Figure S2C), confirming that DNA may mask Clu-binding motifs on histones at the surface of late apoptotic cells. Finally, confocal microscopy revealed a partial colocalization between OG-Clu and histones at the surface of apoptotic cells (Figure 3e).

### Clu is involved in the clearance of apoptotic cells

**Figure 4:**
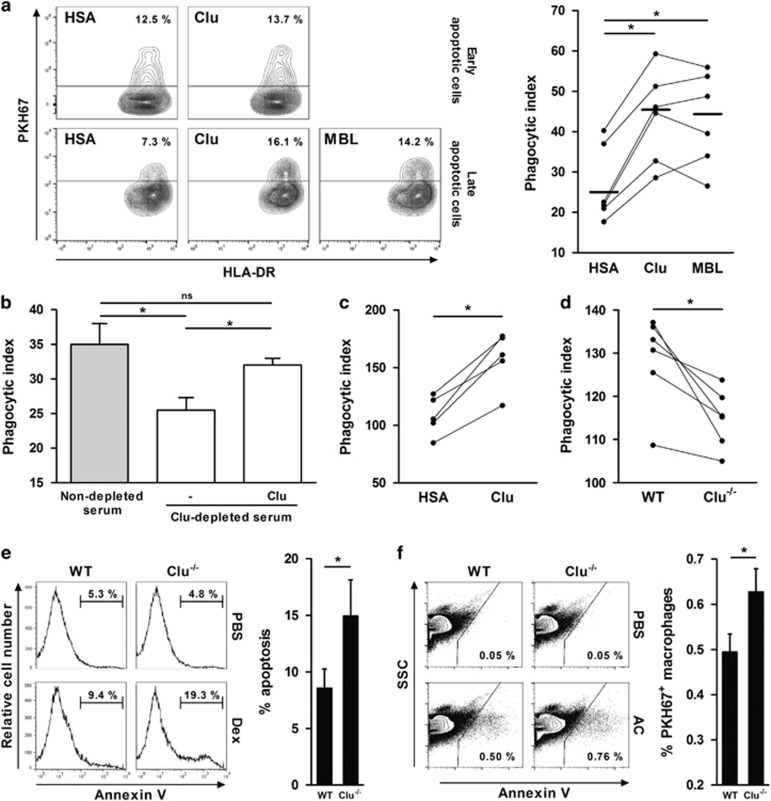
Clu increases apoptotic cells clearance *in vitro* and *in vivo*. (**a**) Mϕ were fed for 30 min with PKH67-labeled early (upper panels) or late apoptotic neutrophils (lower panels) (1 : 10 ratio) previously opsonized or not with 1 μM Clu, MBL, or HSA before incubation with APC-labeled anti-HLA DR mAb. Fluorescence was analyzed by flow cytometry. Left, contour plots are representative of two (upper panels) or six (lower panels) independent experiments; values correspond to the percentage of Mϕ having engulfed dying cells. Right, results of phagocytosis assays using late apoptotic cells are expressed as a phagocytic index (see Materials and Methods); individual determinations are plotted, means are represented by horizontal bars. *P≤0.05. (**b**) Mϕ were fed with PKH67-labeled late apoptotic neutrophils previously incubated in the presence of 30% (v:v) human AB^+^ serum, depleted or not in Clu, and supplemented or not with 1 μM Clu. Results are shown as a phagocytic index, mean±S.E.M.; *n*=4. *P≤0.05; NS, not significant. (**c**) BMDM were fed with PKH67-labeled late apoptotic murine thymocytes (1 : 5 ratio) previously opsonized with 1 μM Clu or HSA before incubation with PE-labeled anti-F4/80 mAb. Fluorescence was analyzed by flow cytometry. (**d**) PKH67-labeled late apoptotic thymocytes were incubated with 1% serum from WT or Clu^−/−^ mice. The phagocytosis assay was performed as previously described. (**e**) Clu^−/−^ and WT mice (*n*=5) were injected intraperitoneally with Dex or with PBS. After 24 h, the frequency of Ann V^+^ apoptotic cells in the thymus was determined by flow cytometry. Left, representative histograms are shown. Values correspond to the percentage of apoptotic cells. Right, results are expressed as the percentage of apoptotic cells, mean±S.E.M., *n*=4; *P≤0.05. (**f**) PKH67-labeled late apoptotic thymocytes (1 × 10^8^) or PBS were injected intravenously in WT and Clu^−/−^ mice. The frequency of PKH67-positive events within splenocytes 2 h after injection was evaluated by flow cytometry. Left, representative dot plots are shown. Values correspond to the percentage of PKH67-positive cells. Right, results are shown as the percentage of the PKH67-positive cells, mean±S.E.M., *n*=4; *P≤0.05

Opsonins act as bridging molecules to favor apoptotic cell clearance. We therefore examined whether Clu may be involved in apoptotic cell clearance using a FACS-based *in vitro* apoptotic cell engulfment assay.^42, 43^ Macrophages (Mϕ) were fed with PKH67-labeled early or late apoptotic neutrophils, previously incubated or not with Clu, MBL, or HSA. Compared with the control protein HSA, Clu enhanced the phagocytosis of apoptotic cells (69±12% increase; mean±S.E.M., *n*=6) in a similar manner to MBL (67±14% increase) (Figure 4a) used as a positive control.^25^ In agreement with the absence of binding to early apoptotic cells, Clu did not modulate the efferocytosis of AnnV^+^ PI^−^ cells (Figure 4a). In order to investigate the role of seric Clu, late apoptotic neutrophils were incubated with heat-inactivated human serum, either depleted or not in Clu. Depletion of Clu reduced the phagocytosis of apoptotic cells (26±6% decrease; mean±S.E.M., *n*=5); this inhibition was partially reversed by supplementing Clu-depleted serum with exogenous Clu (Figure 4b).

Prior to analyzing the role of Clu in the *in vivo* clearance of apoptotic cells, we confirmed the ability of Clu to promote the *in vitro* phagocytosis of apoptotic murine cells. Results showed that (i) late apoptotic thymocytes opsonized with Clu are more efficiently internalized by Mϕ than apoptotic cells incubated with HSA (46±7% increase; mean±S.E.M., *n*=5; Figure 4c), and (ii) that apoptotic thymocytes incubated with serum from Clu^−/−^ mice were less efficiently engulfed by Mϕ than apoptotic cells incubated with serum from wild-type (WT) mice (12±2% decrease; mean±S.E.M., *n*=6; Figure 4d). The *in vivo* role of Clu in apoptotic cell clearance was investigated using Clu^−/−^ mice. In a first set of experiments, we compared, in WT and Clu^−/−^ mice, the clearance of dying thymocytes in which apoptosis was induced by dexamethasone sodium phosphate (Dex).^22, 44^ Remarkably, the thymus of Dex-injected Clu^−/−^ mice contained approximately twofold more remnant apoptotic cells than Dex-injected WT mice (15±3% *versus* 8±2% mean±S.E.M., *n*=5; Figure 4e); we excluded that this observation may result from an increased sensitivity of thymocytes from Clu^−/−^ mice to Dex-induced apoptosis (Supplementary Figure S3A). In contrast, no difference was observed in Clu^−/−^ and WT mice injected with PBS (Figure 4e). In a second set of experiments, we analyzed the splenic clearance of PKH67-labeled apoptotic thymocytes injected intravenously in Clu^−/−^ and WT mice. Two hours after injection, the spleens from Clu^−/−^ mice contained more apoptotic cells than WT mice (0.56±0.06% *versus* 0.44±0.03% mean±S.E.M., *n*=4; Figure 4f); this defect was maintained 6 h after apoptotic cell injection (data not shown). Importantly, Mϕ from Clu^−/−^ mice do not exhibit any defect in apoptotic cell phagocytosis (Supplementary Figure S3B).

### Clu enhances CD4^+^ and CD8^+^ T-cell responses to an apoptotic cell-associated antigen

**Figure 5:**
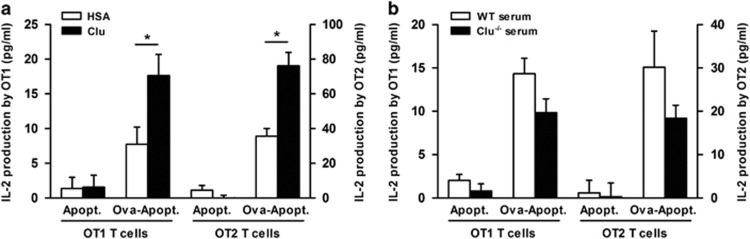
Clu enhances the presentation of apoptotic cell-associated antigen. Thymocytes from Swiss mice were pulsed or not with 35 μM Ova and induced to die by serum deprivation. Apoptotic thymocytes pulsed with Ova (Ova-Apopt) or not (Apopt) were incubated for 30 min (**a**) with Clu or albumin or (**b**) with 10% serum from WT or Clu^−/−^ mice before culture with BMDCs and OT1 or OT2 cells. IL-2 was quantified in the 24-h supernatants by enzyme-linked immunosorbent assay. Results are expressed in pg/ml, mean±S.E.M.; *n*=6; *P≤0.05

The engulfment of apoptotic cells by phagocytes leads to the presentation of apoptotic cell-derived antigens, a process contributing to the maintenance of peripheral tolerance.^45, 46, 47^ We therefore analyzed whether Clu might promote apoptotic cell antigen presentation to CD4^+^ and CD8^+^ T cells. Murine thymocytes were loaded with ovalbumin (Ova) prior to apoptosis induction (Ova-Apopt). In a first set of experiments, dendritic cells (DCs) were incubated with Ova-Apopt previously incubated with Clu or HSA, before culture with Ova-specific OT1 CD8^+^ or OT2 CD4^+^ T cells. Results showed that the opsonization of Ova-Ova-Apopt with Clu enhanced the production of IL-2 by OT1 and OT2 T cells, compared with Ova-Ova-Apopt incubated with HSA (200±64% and 121±19% increase, respectively; mean±S.E.M., *n*=6; Figure 5a). In a second set of experiments, DCs were incubated with Ova-Ova-Apopt previously incubated with 10% serum from Clu^−/−^ or WT mice before culture with Ova-specific T cells. The levels of IL-2 produced by OT1 and OT2 T cells were lower with Ova-Ova-Apopt incubated with serum from Clu^−/−^ mice *versus* serum from WT mice (32±8% and 34±9% decrease, respectively; mean±S.E.M., *n*=4; Figure 5b).

### Clu-deficient mice are sensitive to apoptotic cell-induced autoimmunity

**Figure 6:**
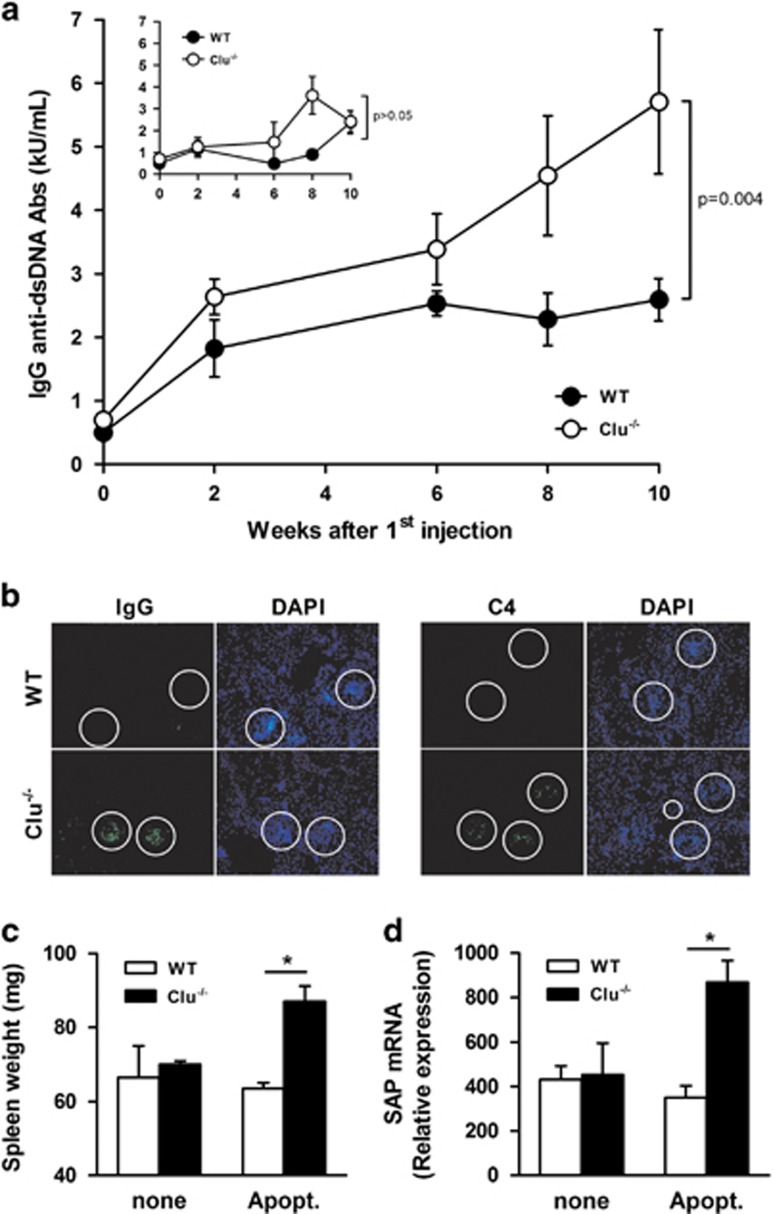
Clu-deficient mice develop autoimmune symptoms in response to apoptotic cells. Aged-matched Clu^−/−^ and WT mice were injected with apoptotic cells once a week for 5 weeks. Two weeks after the first injection of apoptotic cells, 4–6 mice per group were killed bimonthly. (**a**) Circulating IgG anti-dsDNA antibody levels were quantified. Results are expressed in kU/ml, mean±S.E.M., *n*=4 to 10; *P≤0.05. Insert, IgG anti-dsDNA antibody titers were quantified by enzyme-linked immunosorbent assay in the serum of PBS-injected Clu^−/−^ and WT mice. (**b**) Ten weeks after the first injection of apoptotic cells, kidney sections from Clu^−/−^ and WT mice were stained with FITC-labeled anti-mouse IgG (left pictures) or unlabeled anti-C4 revealed with a FITC-labeled anti-rat IgG antibodies (right pictures). Glomeruli were stained with DAPI (circles). Results are representative of four mice. (**c**) Spleens of WT and Clu^−/−^ mice were weighed 10 weeks after the first injection of apoptotic cells or PBS. Results are expressed in mg, mean±S.E.M., *n*=4; *P≤0.05. (**d**) The expression of the mRNA encoding SAP was analyzed by quantitative PCR in the livers from Clu^−/−^ and WT mice 10 weeks after the first injection of apoptotic cells or PBS. Results are expressed as a relative expression with GAPDH used as a calibrator, mean;±S.E.M., *n*=5; *P<0.05

A defect in apoptotic cell clearance may trigger an autoimmune response.^27, 44, 48^ We thereby postulated that the absence of Clu might predispose mice to apoptotic cell-induced autoimmunity. We compared, in Clu^−/−^ and WT mice, the appearance of signs of autoimmunity in a model of mild autoimmune response induced by repeated injections of apoptotic cells.^47, 49^ Results showed that, 2 weeks after the first injection of apoptotic cells, the levels of IgG anti-dsDNA Ab were increased in Clu^−/−^ mice compared with WT mice (2638 U/ml±282 *versus* 1823 U/ml±450, respectively; Figure 6a). In contrast to WT mice, which only developed a slight and transient upregulation 6 weeks after the first injection of apoptotic cells, the levels of IgG anti-dsDNA Abs were significantly higher and maintained elevated in Clu^−/−^ mice, 10 weeks after the first injection of apoptotic cells (Figure 6a). The basal levels of anti-dsDNA Abs remained stable in non-injected Clu^−/−^ mice and were equivalent to the ones in WT mice, although a slight increase was observed as the animal aged (Figure 6a). Interestingly, no difference in the kinetics and amplitude of IgG anti-Ova Ab titers was observed between Clu^−/−^ and WT mice immunized with Ova (Supplementary Figure S4A), suggesting that the induction of anti-dsDNA Abs in Clu^−/−^ mice did not result from an abnormal capacity to mount a humoral response. Interestingly, glomerular IgG and complement component C4 deposits were observed in Clu^−/−^ but not in WT mice, 10 weeks after injection of apoptotic cells (Figure 6b); no deposit was observed in Clu^−/−^ and WT mice injected with PBS (Supplementary Figure S4B). Upon injection of apoptotic cells, the spleen weight of Clu^−/−^ mice was slightly but significantly increased, compared with WT mice (increase of 38±10% mean±SEM, *n*=4), 8 weeks after the first injection of apoptotic cells (Figure 6c). Moreover, 10 weeks after the first injection of apoptotic cells, relative to WT mice, the liver expression of SAP mRNA was enhanced in Clu^−/−^ mice (Figure 6d).

**Figure 7:**
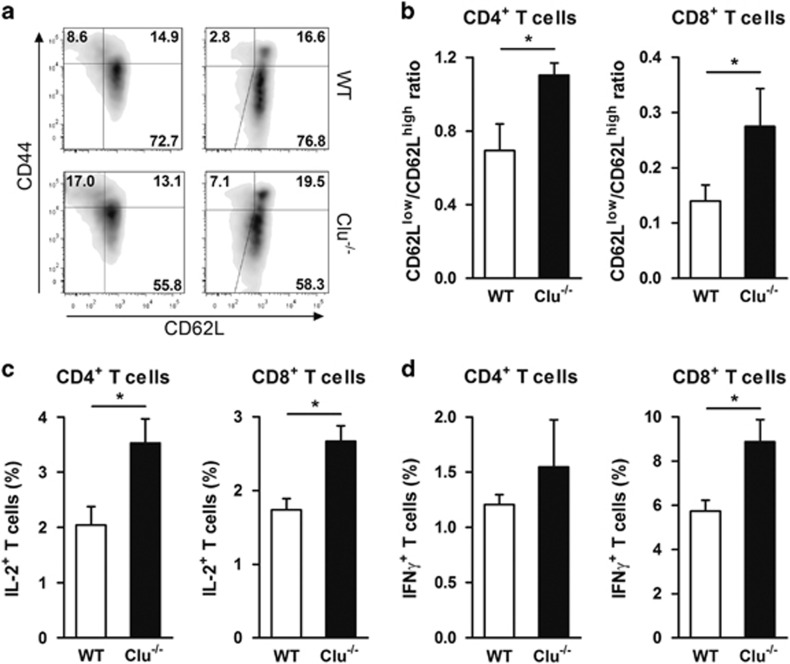
Memory T cells from apoptotic cell-injected Clu-deficient mice exhibit an activated phenotype. Clu^−/−^ and WT mice were injected with apoptotic cells once a week for 5 weeks. Two weeks after the first apoptotic cell injection, 4–6 mice per group were killed bimonthly. The phenotype and function of memory T cells was analyzed 10 weeks after the first injection in Clu^−/−^ and WT mice. (**a**) Lymph node cells were stained with PeCy5-labeled anti-CD3, APCeFluor 780-labeled anti-CD4 or -CD8, FITC-labeled anti-CD62L, and PE-labeled anti-CD44 mAbs. Density plots represent cells gated on the CD3^+^ CD4^+^ (left) and CD3^+^ CD8^+^ (right) populations. Flow cytometric analysis identified CD44^−^ CD62L^high^ naive T cells, CD44^+^ CD62L^high^ central memory T cells, and CD44^+^ CD62L^low^ effector memory T cells. Values correspond to the percentage of each subset. (**b**) CD62L^low^/CD62L^high^ cell ratio among CD3^+^ CD4^+^ CD44^+^ (left histograms) and CD3^+^ CD8^+^ CD44^+^ (right histograms) cells in the lymph nodes of WT and Clu^−/−^ mice. (**c** and **d**) Lymph node cells from Clu^−/−^ and WT mice were stimulated *in vitro* with PMA plus ionomycin in the presence of brefeldin A, before staining with PeCy5-labeled anti-CD3, FITC-labeled anti-CD4, APCeFluor 780-labeled anti-CD8, PE-labeled anti-IL-2, and APC-labeled anti-IFNγ mAbs. Percentages of IL-2- (**c**) and IFNγ-secreting cells (**d**) among CD3^+^ CD4^+^ (left histograms) and CD3^+^ CD8^+^ (right histograms) are shown. (**b**-**d**). Mean±S.E.M., *n*=4 to 6; *P≤0.05

The generation of class-switched IgG autoantibodies in Clu^−/−^ mice suggested the role of T cells. We first analyzed the frequency of naive (CD44^−^ CD62L^high^), central memory (CD44^+^ CD62L^high^), and effector memory (CD44^+^ CD62L^low^)^50^ CD4^+^ and CD8^+^ T cells. Results showed an increase in the frequency of CD44^+^ CD62L^low^ cells within both CD4^+^ and CD8^+^ T-cell subsets, 10 weeks after the first injection of apoptotic cells (Figure 7a). Moreover, the ratio of CD44^+^ CD62L^low^ and CD44^+^ CD62L^high^ cells among CD4^+^ and CD8^+^ T cells were significantly increased in the lymph nodes of Clu^−/−^ (1.10±0.07 and 0.28±0.07, respectively; mean±S.E.M., *n*=5) compared with WT mice (0.70±0.14 and 0.14±0.03, respectively) (Figure 7b). The total numbers of CD4^+^ and CD8^+^ T cells, B cells, Mϕ, and DCs (Supplementary Figure S5A), as well as the frequency of regulatory, memory, naive, and activated T cells, and of activated B cells (Supplementary Figure S5B), were equivalent in the lymph nodes and spleens of non-treated 12-week-old WT and Clu^−/−^ mice. We next determined whether the increased percentage of CD44^+^ CD62L^low^ CD8^+^ and CD4^+^ T cells in apoptotic cell-injected Clu^−/−^ mice had functional implications. Upon stimulation with phorbol myristic acetate (PMA) plus ionomycin, lymph node CD8^+^ and CD4^+^ T cells from Clu^−/−^ mice produced significantly more IL-2 than cells from WT mice (Figure 7c), while only CD8^+^ T cells from Clu^−/−^ mice produced significantly more IFN-γ than cells from WT mice (Figure 7d).

## Discussion

Even though suspected, the potential role of Clu in immune homeostasis remains largely unexplored. We report here that Clu promotes the clearance of late apoptotic cells via its unique capacity to bind to histones translocated to the surface of apoptotic cells. Accordingly, Clu^−/−^ mice develop signs of autoimmunity in a model of apoptotic cell-induced autoimmunity. These results identify Clu as a new bridging molecule involved in the maintenance of tolerance to self-antigens.

After apoptotic cell engulfment, professional antigen-presenting cells (APCs) activate tolerogenic pathways that prevent local inflammatory reactions.^44, 48^ They produce immunoregulatory cytokines (TGFβ, IL-10) and low or no proinflammatory cytokines and chemokines.^51^ In this immunoregulatory environment, the presentation and cross-presentation of apoptotic cell antigens by APCs maintain peripheral T-cell tolerance.^47, 52^ In contrast, in the absence of prompt clearance, apoptotic cells may evolve into immunologically harmful secondary necrotic cells which release danger signals that may favor the initiation of an autoimmune response.^27, 53^ Necrotic cells also trigger the production of inflammatory mediators by APCs.^54^ A rapid and efficient efferocytosis is thus required to maintain immune tolerance. In this study, we demonstrate that Clu, via its unique property to potentiate efferocytosis, prevents the *in vivo* generation of necrotic cells and thereby contributes to maintain self-tolerance.

Despite the loss of billions of cells each day, the incidence of histologically detectable apoptotic cells is rare in normal tissues because of the efficiency of efferocytosis.^22, 44^ We showed that Clu binds specifically to late apoptotic cells, suggesting that, under normal physiological conditions, Clu will have only a minor role in apoptotic cell clearance. Accordingly, Clu^−/−^ mice do not exhibit spontaneous signs of autoimmunity, as observed for most opsonin-deficient mice. Aged MBL-deficient mice do not develop autoimmunity even on a lupus-prone genetic background 129 × C57BL/6.^55^ Moreover, SAP^−/−^ and C1q^−/−^ mice only develop autoimmunity on the mixed 129 × C57BL/6 or MRL/Mp background.^30, 31^ To our knowledge, MFG-E8^−/−^ mice are the only opsonin-deficient model that spontaneously develop an autoimmune phenotype with aging.^29^

The role of Clu in efferocytosis suggests the existence of endocytic receptor(s) for Clu. Megalin was described as a receptor for Clu involved in the uptake of Clu-associated misfolded proteins at the cerebral vascular endothelium and choroid epithelium^56^ and in the endocytosis of cellular debris by epithelial cells.^57^ In humans, however, the expression of megalin is restricted to the proximal tubule of the kidneys, the choroid plexus epithelium, and ependymal cells lining the brain ventricules,^58^ making it unlikely to represent a major endocytic receptor for Clu-mediated efferocytosis by phagocytes. Accordingly, we failed to detect megalin by human Mϕ (unpublished observations). Recent studies have reported that Clu binds to some scavenger receptors^5^ and DC-SIGN,^59^ suggesting that these endocytic receptors may be involved in the internalization of Clu-opsonized apoptotic cells. Experiments are in progress to identify Clu-binding elements involved in the capture of late apoptotic cells by phagocytes.

We have observed that Clu binds specifically to blebs on late apoptotic cells, as reported for other opsonins, such as CRP and SAP.^60^ The fact that the binding of Clu is not dependent on the cell type nor on the apoptosis-inducing method suggested that the Clu-binding motifs are conserved molecules. We demonstrate here that Clu binds to histones. Previous studies have shown that core histone subunits rapidly accumulate in the cytoplasm of early apoptotic cells^61, 62^ before accumulation on blebs.^36, 63^ Accordingly, we observed that histones are expressed by late but not early apoptotic cells, explaining the lack of binding of Clu to early apoptotic cells. These results confirm the role of histones as 'eat-me' molecules at the surface of late apoptotic cells.

Defects in apoptotic cell clearance and/or an excess of apoptotic cells make mice and humans susceptible to autoimmunity.^28^ Although efferocytosis is mediated by multiple and partly redundant mechanisms to avoid the initiation of an autoimmune response, it has been reported that repeated injections of apoptotic cells may induce signs of autoimmunity (without clinical signs).^49^ Considering the role of Clu in efferocytosis, we hypothesized that an excess of apoptotic cells could be less efficiently cleared in Clu^−/−^ mice, leading to a more intense immune response. Indeed, we observed that Clu^−/−^ mice are more sensitive to apoptotic cell-induced autoimmunity. They develop signs of autoimmunity, such as immunoglobulin and complement component C4 deposition within kidneys, autoantibody production, and splenomegaly. In parallel, we observed an activation of effector memory apoptotic cell antigen-specific T cells in Clu^−/−^ mice. In line with these results, previous studies reported that, in models of autoimmune pancreatitis and myocarditis, Clu^−/−^ mice develop more severe inflammatory lesions than WT mice.^14, 15^ However, the mechanism(s) involved in this protective role of Clu was not investigated. Considering our results, it is likely that, in these models associated with a massive cell death, the absence of Clu may have contributed to initiate apoptotic cell-driven autoimmunity.

In conclusion, we show that Clu is a non-redundant opsonin critically involved in the efferocytosis of late apoptotic cells and the maintenance of immune homeostasis. *In vivo*, Clu deficiency leads to a striking autoimmunity induced by the injection of apoptotic cells, a model that mimics a massive cell death that can occur during severe tissue injuries. These results also suggest that Clu may have a protective role against the establishment of chronic sterile inflammatory disorders. This study opens new insights into how to induce tolerance to self-antigens in autoimmune diseases and to optimize immunogenic cell death in antitumor immunotherapies.

## Materials and Methods

### Proteins and antibodies

Human Clu was purified from plasma, as previously described.^7^ Recombinant human MBL and human and murine Clu were from Biotechne (Lille, France). HSA, C1q, and FITC-labeled WGA were from Sigma-Aldrich (St. Louis, MO, USA). SAP (Calbiochem, Darmstadt, Germany), CRP (Millipore, Billerica, MA, USA), and H1, H2A, H2B, H3, and H4 histone subunits (New England Biolabs, Ipswich, MA, USA) were from the indicated providers. Proteins were labeled with Oregon green 488 dye (FluoReporter Oregon Green 488 Protein Labeling Kit; Invitrogen Molecular Probes, Carlsbad, CA, USA) or biotinylated (EZ-Link Sulfo-NHS-LC-Biotin Kit; Pierce, Rockford, IL, USA) using the commercial kits. The origins and clone numbers of the mAbs used in this study are listed in Table 1.

### Isolation and generation of human leukocytes

Blood from healthy subjects was obtained from the Blood collection center of Angers (agreement ANG 2003-2). Human peripheral blood mononuclear cells (PBMCs) were isolated by Ficoll-Paque (Amersham Biosciences, Uppsala, Sweden) density-gradient centrifugation. To generate Mϕ, monocytes were purified from PBMC by positive selection using anti-CD14 mAb-coated magnetic beads (Miltenyi Biotech, Bergisch Gladbach, Germany); purity was >99% (data not shown). Purified CD14^+^ monocytes (1 × 10^6^ cells/ml) were differentiated into Mϕ by 5-day culture with 20 ng/ml M-CSF (Biotechne) and 2 ng/ml GM-CSF (CellGenix, Freiburg, Germany) in complete medium (CM), consisting of RPMI 1640 medium containing 2 mM l-glutamine, antibiotics (all from Lonza, Verviers, Belgium), and 10% (v/v) heat-inactivated fetal calf serum (Biowest, Nuaillé, France). After Ficoll-Paque centrifugation, neutrophils were isolated from erythrocytes by 1.5% (w/v) dextran (Amersham Biosciences) density-gradient sedimentation. Contaminating red blood cells were lysed by hypo-osmotic shock. Purity was routinely >98% (data not shown).

### Isolation and generation of murine leukocytes

C57BL/6 mice and Ova-specific T-cell receptor transgenic mice OT1 and OT2 (C57BL/6 background) were from Charles River Laboratories (L'Arbresle, France). Clu^−/−^ mice (C57BL/6 background) were from The Jackson Laboratory (Bar Harbor, ME, USA). Mice were bred and housed in a pathogen-free environment. Experiments were conducted according to institutional guidelines and were approved by the institutional ethics committee of Région des Pays de la Loire (agreement 2009.18).

#### Murine Mϕ and DC generation

Non-myeloid bone marrow cells were removed after incubation of total bone marrow cells with 10 μg/ml anti-CD4, -CD8, -B220, and -I-Ab mAbs for 20 min at 4 °C and then with rabbit complement (Sigma-Aldrich) for 30 min at 37 °C. Bone marrow myeloid precursors were cultured for 7 days in CM containing 50 ng/ml M-CSF (Immunotools, Friesoythe, Germany) or 10 ng/ml GM-CSF (R&D Systems, Abington, UK) to generate bone marrow-derived Mϕ (BMDM) and DCs (BMDCs), respectively. Non-adherent immature DCs were purified at day 5 by positive selection using anti-CD11c mAb-coated magnetic beads (Miltenyi Biotech). BMDM and BMDC populations contained >95% CD11b^+^ F4/80^+^ and CD11c^+^ I-Ab^+^ cells, respectively (data not shown).

#### Murine CD8^+^ and CD4^+^ T-cell purification

CD8^+^ T cells from OT1 mice and CD4^+^ T cells from OT2 mice were isolated from the spleen and lymph nodes using the CD8^+^ T-Cell Isolation Kit II and the CD4^+^ T-Cell Isolation Kit II, respectively, following the manufacturer's instructions (Miltenyi Biotech). Cell purity, determined by staining for CD3, CD4, CD8, and CD11c expression, was >99% (data not shown).

### Induction of cell death

Spontaneous human neutrophil and murine thymocyte apoptosis was induced by incubating cells in RPMI 1640 medium containing 1% FCS. Staining with allophycocyanin (APC)-labeled Ann V (BD Pharmingen, San Diego, CA, USA) and PI (Sigma-Aldrich) allowed to distinguish four cell populations by flow cytometry, corresponding to viable (Ann V^−^ PI^−^), early apoptotic (Ann V^+^ PI^−^), late apoptotic (Ann V^+^ PI^+^) and secondary necrotic cells (Ann V^+/−^ PI^high^).^32^ In some experiments, cell necrosis was induced by incubating cells at 56 °C for 30 min. Apoptosis of the human T-cell line Jurkat (ATCC, Manassas, VA, USA) was induced by a 24-h incubation with 20 μg/ml etoposide (Sigma-Aldrich) or 20 ng/ml anti-FAS mAb (clone CH-11; MBL International, Woburn, MA, USA). Before each experiment, the Ann V/PI staining was assessed to confirm cell apoptosis. *In vivo* apoptosis of cortical thymocytes was induced by injecting mice intraperitoneally with 0.2 mg Dex (Calbiochem) per 25 g body weight.^22, 44^ After 24 h, the level of thymocyte apoptosis was evaluated by flow cytometry, as described above.

### Binding assays

Apoptotic or necrotic cells (1 × 10^5^ cells/well) were resuspended in PBS containing 1% BSA (w/v) and incubated or not with 1 μM OG-labeled human Clu, recombinant Clu (rClu), C1q, or HSA for 20 min at room temperature. The binding of unlabeled Clu was detected with an anti-Clu mAb (Biotechne); mouse IgG1 antibody (R&D Systems) was used as a control. Bound antibodies were detected with FITC-labeled anti-mouse Ig Ab (BD Pharmingen). Fluorescence was analyzed by flow cytometry. In some experiments, apoptotic Jurkat T cells (containing at least 60% Ann V^+^ PI^+^ cells) were treated for 1 h with 500 μg/ml DNase or for 30 min with 100 μg/ml pronase (both from Roche, Mannheim, Germany) or for 4 h with protein deglycosylation mix (New England Biolabs) prior to the binding assay. In other experiments, binding of Clu to 'flip-flopped' erythrocytes^64^ was measured. The binding of Clu, SAP, CRP, and HSA to histone subunits and dsDNA was measured by a solid-phase binding assay. Briefly, 1 μg/ml of each histone subunit was coated on 96-well plates in 50 mM NaHCO_3_/Na_2_CO_3_ buffer, pH=9.6, for 12 h at 4 °C. dsDNA was coated as previously described.^44^ After blocking the nonspecific sites with PBS containing 5% BSA, plates were incubated for 2 h with 1 μg/ml of biotinylated Clu, SAP, CRP, and HSA and then with HRP-labeled streptavidin (Diaclone, Besançon, France) diluted 1 : 500 for 1 h. The coating of dsDNA was verified using an anti-dsDNA Ab (Immunotools) revealed by HRP-conjugated anti-mouse IgG Ab (Life Technologies, Saint Aubin, France). Optical density was read at *λ*=492 nm.

### Phagocytosis assays

#### Phagocytosis assay with human cells

Freshly isolated neutrophils were labeled with the green fluorescent dye PKH67 using the PKH67 Green Fluorescent Cell Linker Kit (Sigma-Aldrich), according to the manufacturer's instructions. Apoptosis was induced as described above. Mϕ (2 × 10^5^ cells/well) were cultured in 48-well plates for 12 h before the assay. PKH67-labeled early (corresponding to a cell population containing no late apoptotic cells) or late apoptotic neutrophils (corresponding to a cell population containing at least 80% late apoptotic cells) were incubated for 30 min in RPMI 1640 medium, containing or not 1 μM Clu, MBL, or HSA. After washing, 1 × 10^6^ neutrophils were added to Mϕ and incubated for 40 min at 37 °C in RPMI 1640 medium. Non-internalized apoptotic cells were removed by washing Mϕ with ice-cold PBS.^32^ Cells were then incubated with an APC-labeled anti-HLA-DR mAb. Phagocytosis was analyzed by flow cytometry, as previously described.^32^ In some experiments, apoptotic neutrophils were incubated for 30 min in RPMI 1640 medium containing 30% human serum, depleted or not in Clu, prior to the phagocytosis assay. Clu-depleted serums and control serums were prepared by passing human serums over a column of anti-Clu mAb or isotype control mAb covalently linked to agarose beads (co-IP columns; Pierce). Depletion was verified by quantifying Clu by ELISA (Biotechne); results showed that 89±2% (mean±S.E.M., *n*=4) of Clu was removed from the serum (not shown).

#### Phagocytosis assay with murine cells

Freshly isolated thymocytes from C57BL/6 mice were labeled with PKH67, prior to apoptosis induction. BMDM (2 × 10^5^ cells/well) were cultured in 48-well plates for 12 h before the assay. Opsonization of apoptotic thymocytes (80% late apoptotic cells) was performed as described above. After washing with ice-cold PBS, BMDM were incubated with phycoerythrin (PE)-labeled anti-F4/80 mAb. In some experiments, apoptotic thymocytes were incubated with 1% (v:v) serum from WT or Clu^−/−^ mice, prior to incubation with BMDM. Results are expressed as a percentage of phagocytosis (percentage of PKH67^+^ events among the HLA-DR^+^ or F4/80^+^ populations) or as a phagocytic index determined as follows: (percentage of phagocytosis × MFI of double-positive events)/100.^63^

#### In vivo phagocytosis assay

Thymocytes from WT mice were labeled with PKH67 and apoptosis was induced as described above. Apoptotic thymocytes (1 × 10^8^ cells containing at least 80% late apoptotic cells) were injected intravenously into Clu^−/−^ and WT mice. The frequencies of PKH67-labelled cells among splenocytes were analyzed by flow cytometry 2 h after injection.

### *In vitro* activation of OT1 and OT2 cells

Ova (Affiland, Liege, Belgium) and BSA were first dialyzed and detoxified (EndoTrap system; Profos, Regensburg, Germany) before use. Thymocytes from outbred Swiss mice were pulsed with 35 μM Ova or BSA by osmotic shock, as previously described,^50, 65^ prior to apoptosis induction. Apoptotic thymocytes (containing at least 80% late apoptotic cells) were incubated or not with albumin or murine rClu, in RPMI 1640 medium. After washing, 5 × 10^5^ apoptotic thymocytes were co-cultured with 1 × 10^5^ BMDCs and 1 × 10^5^ OT1 or OT2 cells. After 24 h, the production of IL-2 was monitored by ELISA (BD Pharmingen).

### Laser confocal scanning microscopy

Human Mϕ were cultured on glass slides in 48-well plates, 24 h prior to the phagocytosis assay. After phagocytosis, Mϕ were washed with ice-cold PBS, fixed in 2% (w/v) paraformaldehyde (PFA) in PBS and stained with a PE-labeled anti-CD14 mAb. Slides were then mounted with ProlonGold reagent (Invitrogen). In some experiments, apoptotic neutrophils were incubated with OG-Clu as described above and fixed with 2% PFA in PBS. In others, apoptotic neutrophils were stained with anti-H2/H3/H4 mAb; bound mAb were detected with a PE-labeled anti-mouse Ig Ab (Dako, Glostrup, Denmark). Cells were then mounted on glass slides with ProlonGold reagent after nucleus staining with DAPI (Invitrogen). Staining was analyzed with a confocal laser scanning system (A1Rsi, Nikon, Tokyo, Japan).

### PCR analysis

The expression of the mRNA encoding SAP was analyzed in the livers from Clu^−/−^ and WT mice. Total RNA, purified using the RNeasy Plus MiniKit (Qiagen, Düsseldorf, Germany) was reverse transcribed using the superscript II Reverse Transcriptase (Invitrogen). mRNA expression was determined by reverse transcriptase-PCR using iQ SYBR Green Supermix (Bio-Rad, Hercules, CA, USA). Specific gene expression was calculated using the 2^−ΔCT^ method (using GAPDH as a calibrator).

### Induction and monitoring of autoimmunity in mice

WT and Clu^−/−^ mice (8-week old) were injected intravenously with 1 × 10^7^ irradiated apoptotic cells (ATCC), once a week for 5 weeks (from week 1 to week 5). Serum samples were collected once every 2 weeks after the first injection, for up to 10 weeks (at week 2, 4, 6, 8, and 10). Serum anti-dsDNA IgG Ab were quantified by ELISA (Alpha Diagnostic International, San Antonio, TX, USA). Every 2 weeks, 4–6 mice from each group were killed, and the kidneys, spleen, and lymph nodes were recovered. Lymph node cells were stained with PE-Cy5-labeled anti-CD3, APCeFluor 780-labeled anti-CD4 or anti-CD8 mAbs, FITC-labeled anti-CD19, PE-labeled anti-CD44, and FITC-labeled anti-CD62L Abs. In some experiments, lymph node cells were stimulated *in vitro* with 10 ng/ml PMA and 1 μM ionomycin for 6 h in the presence of 10 μg/ml brefeldin A (all from Sigma-Aldrich). Cells were then fixed for 10 min with 4% PFA in PBS and incubated for 30 min in PBS containing 0.1% BSA and 0.1% saponin (both from Sigma-Aldrich) with PeCy5-labeled anti-CD3, FITC-labeled anti-CD4 mAb, APCeFluor 780-labeled anti-CD8, PE-labeled anti-IL-2, and APC-labeled anti-IFNγ mAbs. Fluorescence was analyzed by flow cytometry. Kidneys were frozen, cryosected, and fixed in cold acetone. Tissue sections were stained with FITC-labeled goat anti-mouse IgG antibodies (Dako) or unlabeled rat anti-C4 mAb (Cedarlane, Burlington, Canada). Binding of anti-C4 mAb was detected with a FITC-labeled goat anti-rat Ig Ab (BD Biosciences, San Jose, CA, USA). After washing, DAPI was added and the cells were mounted on glass slides with ProlonGold reagent. Slides were examined using a Leica DMR fluorescence microscope with an IM500 image manager system (Leica, Wetzlar, Germany).

### Statistical analysis

Data are shown as mean±S.E.M. or as a percentage of the increase ((*B*−*A*)/*A*) × 100) or decrease ((*A*−*B*)/*A*) × 100), where *A* is the control value and *B* the value obtained with the protein of interest, mean±S.E.M. Data were analyzed by the one-tailed Wilcoxon matched-pairs test or by the one-tailed Mann–Whitney test or by two-way ANOVA test. *P*≤0.05 was considered statistically significant.

## Figures and Tables

**Table 1:** List of monoclonal antibodies (mAb) used

**mAb**	**Clone**	**Reactivity**	**Provider**
Anti-B220	RA3-6B2	Mouse	BD Pharmingen
Anti-CD19	MB19-1	Mouse	eBioscience
Anti-CD3	145-2C11	Mouse	eBioscience
Anti-CD4	L3T4	Mouse	eBioscience
Anti-CD8	53-6.7	Mouse	eBioscience
Anti-CD44	IM7	Mouse	BD Pharmingen
Anti-CD11b	M1/70	Mouse	BD Pharmingen
Anti-CD11c	N418	Mouse	eBioscience
Anti-CD14	TUK4	Human	Dako
Anti-CD19	MB19-1	Mouse	eBioscience
Anti-CD45RA	HI100	Mouse	BD Pharmingen
Anti-CD62L	MEL-14	Mouse	eBioscience
Anti-Clu	350270	Human	R&D Systems
Anti-dsDNA	HpS22	Human/Mouse	Immunotools
Anti-F4/80	BM8	Mouse	Invitrogen
Anti-H2/H3/H4	HB-9	Human/Mouse	AbD Serotec
Anti-H2-Kb	AF6-88.5	Mouse	BD Pharmingen
Anti-H4	F-9	Human/Mouse	Santa Cruz Biotech
Anti-HLA-DR	L243	Human	BD Pharmingen
Anti-IAb	AF6-120.1 & 25-9-3	Mouse	BD Pharmingen
Anti-IFNγ	4S.B3	Mouse	eBioscience
Anti-IL-2	JES6-5H4	Mouse	eBioscience

## Abbreviations

Ann V: annexin V
APC: antigen-presenting cells
Clu: clusterin
CRP: C-reactive protein
HSA: human serum albumin
Mϕ: macrophages
MBL: mannose-binding lectin
Ova: ovalbumin
PI: propidium iodide
PRR: pattern recognition receptor
RA: rheumatoid arthritis
SAP: serum amyloid P component
