# Supplementary figures and images for: Clusterin facilitates apoptotic cell clearance and prevents apoptotic cell-induced autoimmune responses
Source: Cell Death Dis. 2016 May 5;7(5):e2215–. doi: 10.1038/cddis.2016.113 (PMC4917652; doi:10.1038/cddis.2016.113)

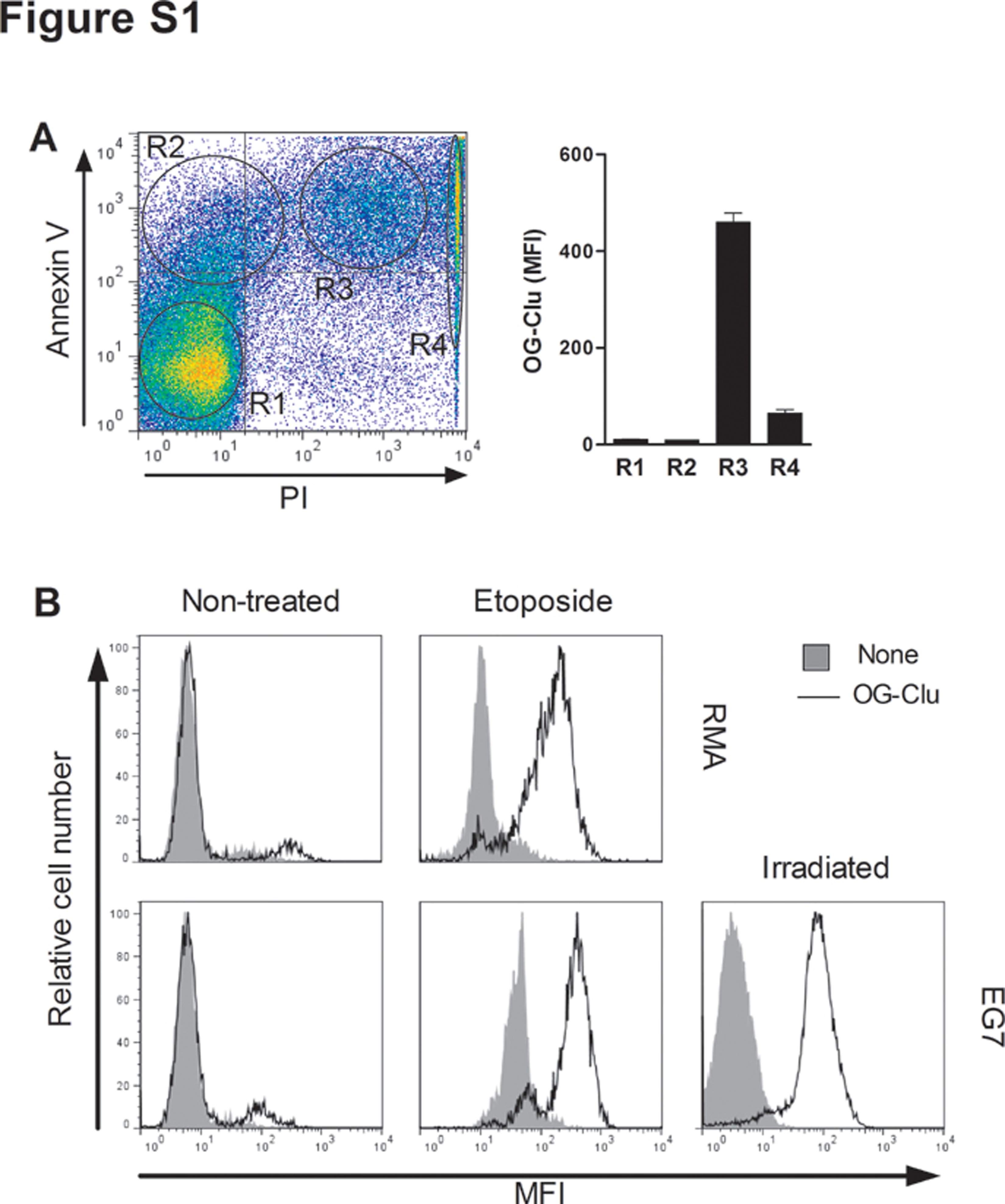

Supplement: Supplementary Figure 1 [file cddis2016113x1.tif]

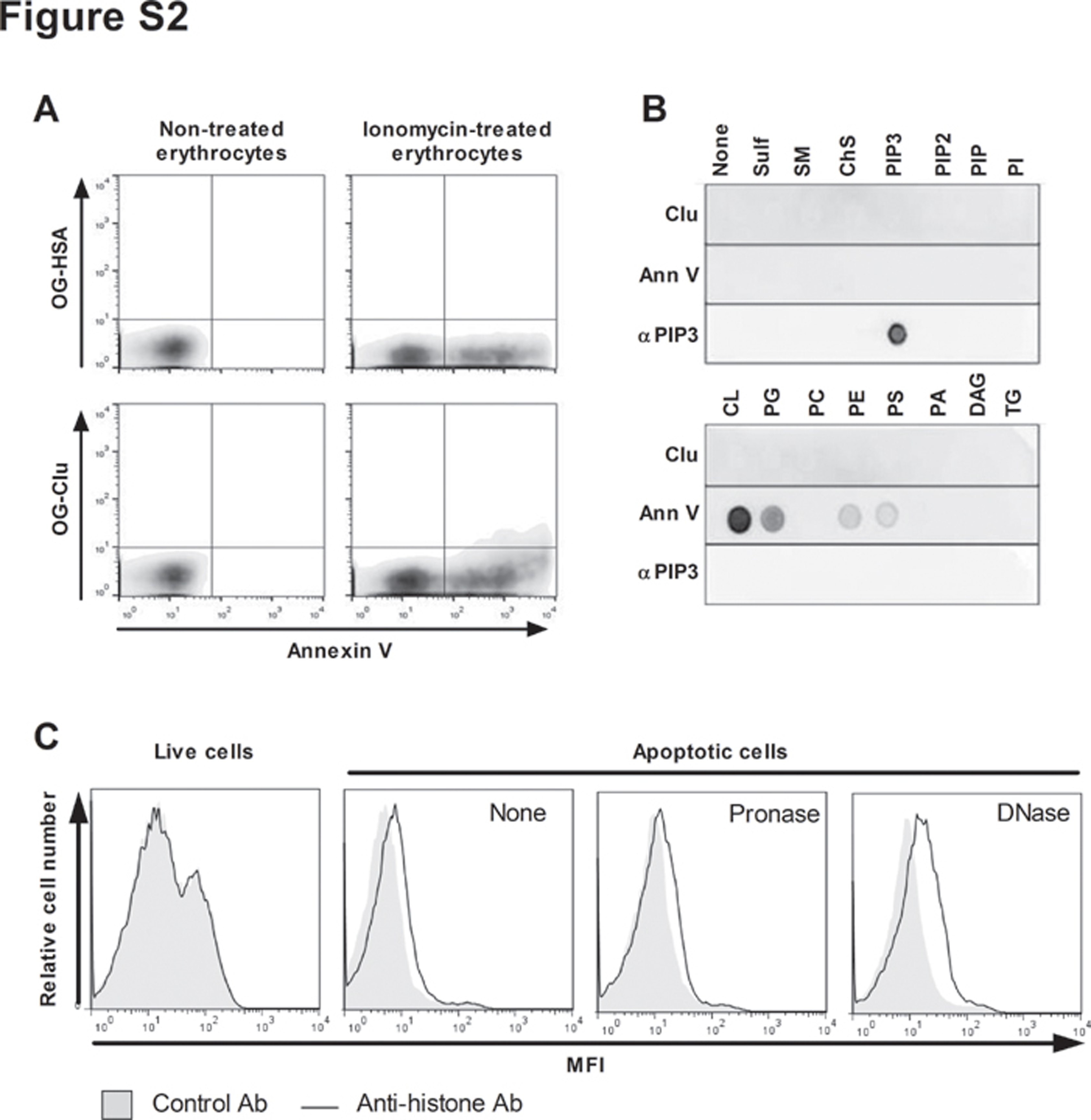

Supplement: Supplementary Figure 2 [file cddis2016113x2.tif]

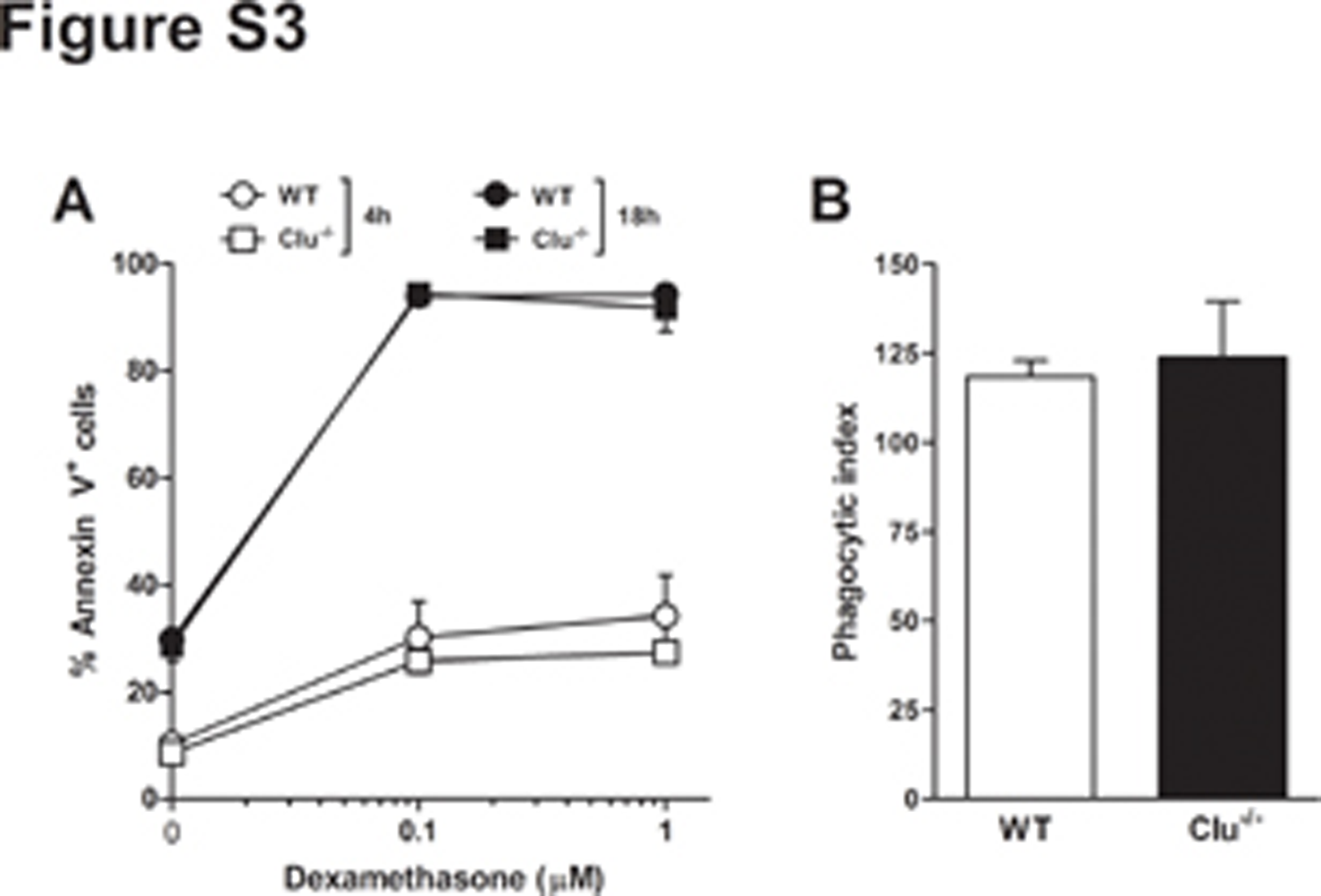

Supplement: Supplementary Figure 3 [file cddis2016113x3.tif]

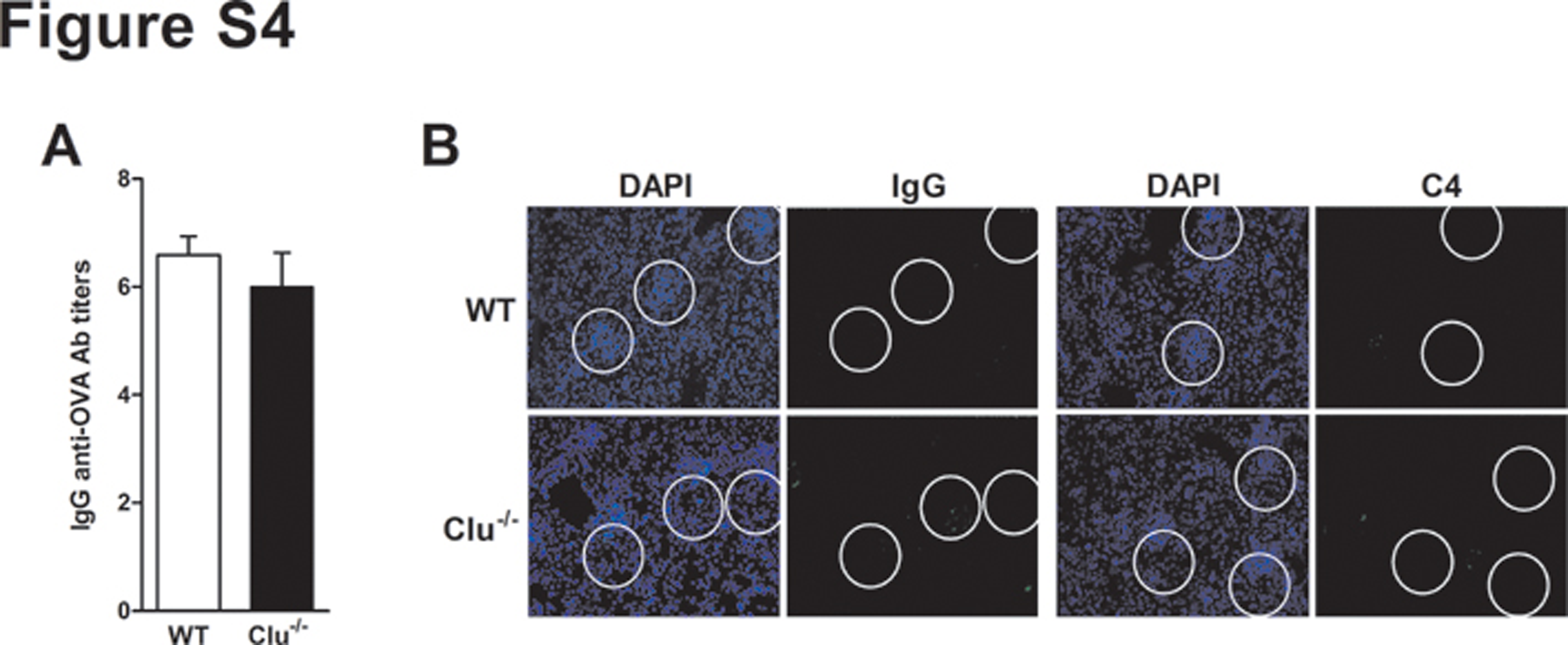

Supplement: Supplementary Figure 4 [file cddis2016113x4.tif]

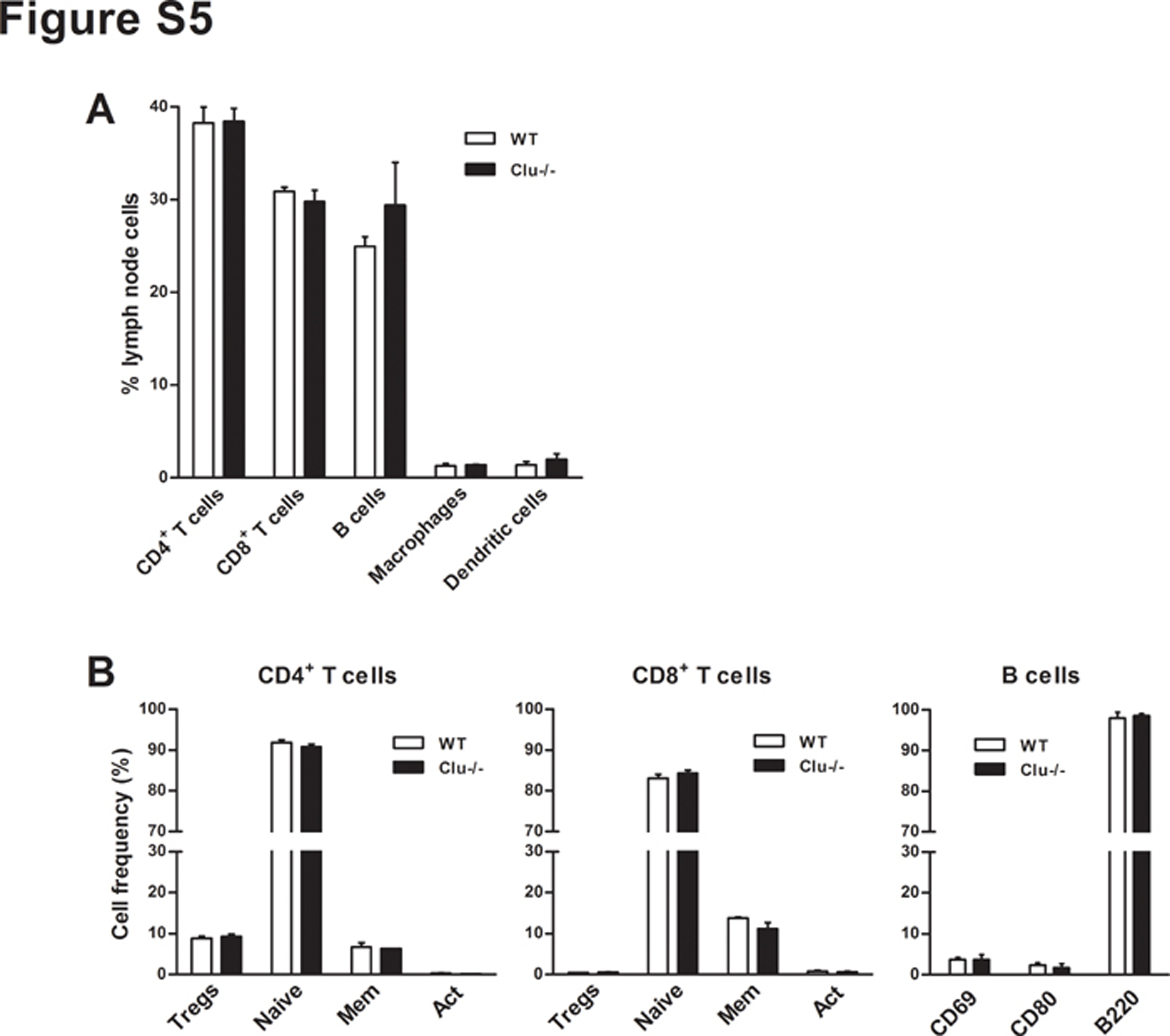

Supplement: Supplementary Figure 5 [file cddis2016113x5.tif]
