# Supplementary material for: Clusterin facilitates apoptotic cell clearance and prevents apoptotic cell-induced autoimmune responses
Source: Cell Death Dis. 2016 May 5;7(5):e2215–. doi: 10.1038/cddis.2016.113 (PMC4917652; doi:10.1038/cddis.2016.113)
Supplement: Supplementary Figure Legends [file cddis2016113x6.doc]

**Supplemental Figure legends**

**Supplemental Figure 1**. **A**. Left panel, apoptosis of thymocytes was induced by serum deprivation, and assessed by PI and Ann V staining. Flow cytometric analysis identified 4 populations (R1-R4). Right panel, analysis of the binding of OG-Clu to R1-R4 populations results are expressed as MFI values, mean ± SEM, n=4. **B**. Flow cytometry histograms showing the binding of OG-Clu to apoptotic RMA (ATCC, Manassas, VA) (upper panels) and EG7 (lower panels) cells, untreated or treated with 20 µg/ml etoposide (RMA and EG7 cells), or irradiated (EG7 cells). Histograms are representative of 4 independent experiments.

**Supplemental Figure 2.** **A**. Human erythrocytes were untreated or treated with ionomycin (to induce membrane flip-flopping) prior addition of OG-Clu or OG-HSA. Externalization of phosphatidyl serine, evidenced by Ann V staining, and binding of OG-Clu and OG-HSA were analyzed by flow cytometry. Density plots are representative of three independent experiments. **B**. Membrane lipid strips (Echelon Biosciences, Salt Lake City, UT) were incubated with biotinylated-Clu, -Ann V or –anti PIP3 mAb (Echelon Biosciences). Bound Clu, Ann V, or anti-PIP3 were detected using the ECL system (GE Healthcare, Bucking-hamshire, U.K.). Result is representative of two independent experiments. **C.** Jurkat T cells were untreated or treated with 20 μg/ml etoposide. Before treatment with pronase or DNase, as described in the Materials and Methods section. Live and apoptotic Jurkat cells were labeled with anti-H2/H3/H4 mAb or isotype control Ab, before incubation with FITC-labeled anti-mouse IgG Ab. The presence of histones on the cell surface was assessed by flow cytometry. Results are representative of two independent experiments.

**Supplemental Figure 3**. **A**. Freshly isolated murine thymocytes from Clu^-/-^ and wild type mice (1.10^6^ cells/well) were incubated for 4 or 18 h with the indicated concentrations of Dex. Apoptosis was assessed by Ann V staining. **B**. BMDM were generated from Clu^-/-^ and WT mice. The phagocytosis assay was performed as described in the Materials and Methods section. Results are expressed as a phagocytosis index, mean ± SEM; n=3.

**Supplemental Figure 4**. **A**. Wild type and Clu^-/-^ mice were subcutaneously injected with incomplete Freund’s adjuvant and OVA at day 0 and day 15. Anti-OVA IgG Ab were quantified by ELISA at day 30. IgG titers were normalized to those obtained in PBS-injected mice, and are expressed as anti-OVA IgG Ab titers (mean ± SEM, n=4 to 7). **B**. Kidney sections from Clu^-/-^ and WT mice collected 10 weeks after the first PBS injection, were stained with FITC-labeled anti-mouse IgG Ab (left pictures), or with unlabeled anti-C4 Ab revealed with a FITC-labeled anti-rat Ig Ab (right pictures), and DAPI. Dotted circles represent glomeruli. Results are representative of 4 mice.

**Supplemental Figure 5**. **A**. Frequencies of CD3^+^ CD4^+^ T cells, CD3^+^ CD8^+^ T cells, CD19^+^ B cells, F4/80^+^CD11b^+^ Mϕ and F4/80^-^CD11c^+^ DC among lymph node cells were analyzed. Results are expressed in percentages of total lymph node cells (mean ± SEM, n=3). **B**. T cells include: Tregs (CD25^+^ FoxP3^+^), Naive (CD44^-^ CD62L^+^), Mem (memory, CD44^+^ CD62L^-^), and Act (activated, CD44^+^ CD62L^-^) CD4^+^ and CD8^+^ T cells; B cells include activated CD19^+^ CD69^+^, CD19^+^ CD80^+^, and CD19^+^ B220^+^ (right panel). Results are expressed in percentages of total CD3^+^ CD4^+^ (left panel), CD3^+^ CD8^+^ (middle panel), and CD19^+^ cell (right panel) populations (mean ± SEM, n=3).
